# Antibiotic and Surgical Treatment of Diabetic Foot Osteomyelitis: The Histopathological Evidence

**DOI:** 10.3390/antibiotics13121142

**Published:** 2024-11-27

**Authors:** Roberto Da Ros, Roberta Assaloni, Andrea Michelli, Barbara Brunato, Cesare Miranda

**Affiliations:** 1 Diabetes and Diabetic Foot Treatment Center, Monfalcone-Gorizia, ASUGI, 34074 Monfalcone, Italy; 2 Clinic of Endocrinology and Metabolism Diseases, ASFO, 33170 Pordenone, Italy; cesare.miranda@asfo.sanita.fvg.it

**Keywords:** diabetic foot, osteomyelitis, antibiotics, margin

## Abstract

**Background:** Osteomyelitis is one of the most frequent infections of the diabetic foot, accounting for 20–70% of foot infections. The treatment of osteomyelitis continues to be debated, and the possibility of performing conservative surgery associated with targeted antibiotic treatment allows for reductions in the amount of bone removed, the resolution of osteomyelitis, and a reduction in the changes in the biomechanics of the foot. The objective of this study was to evaluate the outcomes of osteomyelitis treatment with a combination of antibiotic and surgical procedures based on a histopathological analysis of the infected bone and margins. **Materials and Methods:** We analyzed 25 diabetic patients with osteomyelitis. We treated each patient with empiric antibiotic treatment, surgical removal of the infected bone, and targeted antibiotic treatment. During the surgical procedure, we collected infected bone samples and margins for microbiological and histopathological analyses. **Results:** All the patients had type 2 diabetes, with a mean age of 71 ± 10 years. Antibiotic therapy was administered orally for an average duration of 21 ± 9 days, aimed at improving the microbiological outcome. Histological examinations of the resected infected bone revealed the presence of osteomyelitis in 23 (92%) patients. The healthy margin sample, surgically assessed as non-infected, was confirmed negative in 80% of cases. At a follow-up of 18 ± 7 months, we achieved complete healing in twenty patients (80%), with an average healing time of 70 ± 41 days. No recurrence of osteomyelitis was observed. **Conclusions:** The data from this study demonstrate that the combination of targeted antibiotic therapy and conservative surgical treatment is effective in resolving osteomyelitis without recurrence with a very long follow-up. Histological analyses allowed us to confirm the actual presence of osteomyelitis and demonstrate that clinical differentiation during surgery is effective in identifying a healthy margin.

## 1. Introduction

It is estimated that more than 500 million people worldwide are affected by diabetes mellitus [1].

Diabetes-related foot infections (DFIs) are among the most frequent, serious, and costly complications of diabetic disease [2]. Between 10 and 20% of moderate DFIs and between 40 and 60% of severe DFIs are complicated by diabetes-related foot osteomyelitis (DFO) [3,4]. Diabetic foot osteomyelitis (DFO) is an infection of the bone with involvement of the marrow, according to the definition recently revised by the International Working Group on the Diabetic Foot (IWGDF) [5]. This DFO complication significantly increases the risk of lower-limb amputation [6]. Bone infection is believed to occur through the direct spread of contaminating organisms from an ulcer, rather than hematogenously [7].

The diagnosis of DFO is not always simple, as the clinical signs and symptoms are not always specific [8]. There are several diagnostic tools and criteria, such as the probe-to-bone test, serum biomarkers, and plain X-rays [9,10,11]. The definitive diagnosis of osteomyelitis of the foot requires the collection of bone samples for microbiological and histopathological studies [11], but few data are available on the histological characteristics of DFO [12].

According to Aragon-Sanchez et al., we can find three different histomorphological anomalies in DFO: acute (destroyed bone and infiltrations of polymorphonuclear granulocytes at the cortical sites and within the bone marrow usually associated with congestion or thrombosis of small medullary or periosteal vessels); chronic (bone destruction and lymphocytic infiltration of histiocytes and/or plasma cells in cortical sites and within the bone marrow); and acute exacerbation of chronic osteomyelitis (a background of chronic osteomyelitis, in which there is an infiltration of polymorphonuclear granulocytes). In all cases, areas of fibrosis and bone marrow edema may occur [13].

The treatment of DFO typically involves a multidisciplinary approach that includes antimicrobial therapy, surgery, and the management of underlying diabetic complications [2]. In recent years, the treatment of osteomyelitis in the diabetic foot has continued to be debated. Although surgical resection of the infected bone is considered the gold standard of treatment [14,15,16], there have been some studies that have demonstrated the effectiveness of antibiotic treatment [17,18,19,20,21]. Until recently, most experts believed that the standard treatment for diabetic foot osteomyelitis should be surgical removal of the infected bone, while antibiotic treatment alone can achieve remission of osteomyelitis in patients without advanced bone involvement [14,22]. However, the widespread and growing bacterial resistance to antibiotics [23] and the limited penetration of antibiotics into bone are the main reasons why surgery is often preferable to antibiotic treatment.

In recent years, emerging data have underlined the possibility of performing conservative surgery with minimal bone resection, with good effects in resolving osteomyelitis, minimizing the duration of antibiotic therapy, and reducing the change in the biomechanics of the foot [13,24]. However, despite the prevalence, costs, and morbidity of these infections, published evidence demonstrating the effectiveness of these approaches is limited [25]. Some efforts have been made to standardize the sequestration procedure to avoid incomplete bone resection and the recurrence of infection [26,27].

The aim of this study was to evaluate the clinical effectiveness of antibiotics and conservative surgery in the treatment of diabetic foot osteomyelitis by histopathologically verifying the infected bone and the healthy margin.

## 2. Results

We obtained samples from 25 consecutive people with diabetes. All the patients had type 2 diabetes; the mean age was 71 ± 10 years (mean ± SD); and five of the patients were women (20%). The mean HA1c was 7.9 ± 2.1%, the mean creatinine concentration was 1.09 ± 0.5 mg/dL, and the mean BMI was 27.4 ± 3.4 m^2^/kg. All the patients presented with arterial hypertension, 12 (48%) presented with coronary artery disease, and 5 (20%) presented with cerebral vascular disease with a previous history of stroke. As regards lower-limb complications, peripheral neuropathy was present in all the patients, while eighteen (72%) had peripheral vascular disease, and eleven of them required endovascular revascularization before surgery (Table 1).

All lesions were Texas grade 3 (4 grade 3A, 8 grade 3B, 13 grade 3D) and had been present for 184 ± 198 days. In all patients, the diagnosis of osteomyelitis was confirmed by the presence of a positive probe-to-bone test and a positive radiograph (erosion of cortical bone, cortical lysis, osteolysis, bone sequestration). The bone subjected to sequestrectomy was the phalanx in seven cases, metatarsal bone in seventeen cases, and cuboid in the remaining one. In all cases, clinical distinction between the healthy bone and infected bone was possible.

### 2.1. Microbiological Results and Antibiotic Treatment

Antibiotic therapy: before proceeding with the surgery, all patients were started on empiric antibiotic therapy: 60% with amoxicillin/clavulanic, 20% with quinolones, and 20% with piperacillin/tazobactam. Throughout sequestrectomy, a part of the infected bone was taken for microbiological examination, leading to a negative result in 6 (24%) cases and a positive result in 18 cases, with one microorganism and one case that was positive with two bacteria. The most prevalent bacteria were Gram-positive (60%).

We recorded eleven cases of isolated Staphylococcus species (five *St. aureus*, one *St. epidermidis*, four *St. haemolyticus*, one *St. auricolaris*), four of *Pseudomonas aeruginosa*, two of *Citrobacter freundii*, one of *Proteus mirabilis*, one of *Enterococcus faecalis*, and one of *Clostridium sporogenes*.

Antibiotic resistance: *staphylococcus* was methicillin-resistant in nine cases (73%). No *Pseudomonas aeruginosa* resistant to quinolones was found; see Table 2.

After the microbiological analysis, we administered targeted antibiotic therapy: 36% of the patients continued with amoxicillin/clavulanic and 56% with quinolones; the remaining 8% continued with teicoplanin and trimethoprim. Antibiotic therapy was administered for an average duration of 21 ± 9 days (Table 3).

### 2.2. Histological Analysis Results

The histological examination of infected bone samples, considered to be clinically infected bone, confirmed the presence of osteomyelitis in 23 (92%) samples, 19 with the presence of acute inflammation and 4 with a chronic inflammatory component. Two samples were negative for osteomyelitis, and bone with a fibro-productive process without inflammation was present. In the infected bone samples, 76% had neutrophils, 16% eosinophils, 36% microabscesses, and 48% necrosis of the trabeculae. Clinically healthy margin bone samples showed acute inflammation in only 16% of cases, while 84% of cases exhibited chronic inflammation, 16% neutrophils, 4% eosinophils, 4% microabscesses, and 12% necrosis of the trabeculae. The differences between the infected bone and margin were found to be statistically significant for acute inflammation, chronic inflammation, microabscesses, trabecular necrosis, and the presence of neutrophils. Osteoclastic activity was comparable between the two samples. In five (20%) patients, the resection margin considered clinically healthy was positive for acute inflammation and, therefore, considered infected (Table 4).

### 2.3. Outcome

At the follow-up of 18 ± 7.5 months, twenty patients (80%) had complete wound healing with a mean healing time of 70 ± 41 days. Five patients (20%) did not recover with the surgical procedure due to superinfection (one person) or ischemic recurrence (two people) and underwent further procedures; two patients died before recovery. After surgery, the patients were seen as outpatients every 2 weeks and treated with local dressings and lesion offloading.

The five non-healed patients did not present statistically significantly different clinical or microbiological characteristics. Non-healed patients presented a higher level of acute phlogosis in the histologic samples of the infected bone (*p* = 0.02). The antibiotic therapy duration was similar in the healed and non-healed groups (20 ± 8 vs. 26 ± 11, *p* n.s.). The five patients with a positive resection margin had younger age (63 ± 12 vs. 73 ± 8, *p* < 0.05), lower creatinine levels (0.72 ± 0.13 vs. 1.18 ± 0.48, *p* < 0.05), higher frequency of positive microbiological examination (100% vs. 60%, *p* < 0.001), and a significantly higher acute inflammatory component and more microabscesses in the corresponding infected bone sample (*p* < 0.02) compared to patients with a negative margin. The infected margin had significantly lower levels of acute inflammation than the corresponding infected bone (1.4 ± 0.9 vs. 2.6 ± 0.9, *p* < 0.05). No significant differences were found in terms of the time of injury, the percentage and time of healing, or the duration of antibiotic therapy. The time of healing was similar: 63 ± 36 days for the negative margin and 86 ± 51 days for the positive margin (Table 5).

## 3. Discussion

Targeted antibiotic therapy combined with the limited removal of the infected bone in diabetic foot osteomyelitis may ensure the resolution of the infection. In our work with this approach, 80% of patients achieved recovery from osteomyelitis without recurrence after a long follow-up. The remaining 20% of patients not healed with a single surgery underwent further surgical revision or proximalization of the amputation. Our approach involves initial empiric antibiotic therapy, surgical treatment limited to the infected bone, and subsequently targeted antibiotic therapy based on the bone culture obtained during the operation. We also histologically analyzed both the infected bone to confirm the presence of osteomyelitis and the healthy margin to evaluate the effectiveness of the surgical procedure. Histological analysis of the infected bone confirmed that the combination of positive probe-to-bone tests and pathological radiography was able to identify the presence of osteomyelitis with a probability of 92%. The remaining 8% of cases did not have acute inflammation but chronic changes, probably indicative of the resolution of osteomyelitis. Histological analysis of the healthy margin demonstrated that the systematic surgical approach, as described by Schmidt et al., was able to distinguish the infected bone (to be removed) from the healthy bone (to be left in place) [26].

The clinical data present in the literature [13,14] already highlight the effectiveness of surgical removal of the infected bone; however, the histological demonstration was lacking. The clinical efficacy is confirmed by the data of this study with the observation that the histopathological samples demonstrate a clear distinction between the two types of bones taken: infected bone and margin. Signs of acute inflammation, microabscesses, and necrosis of the trabeculae are the elements that characterize the infected bone. The bone margin considered healthy instead has a chronic inflammatory component but absence of bone destruction and acute infection.

To achieve this result, the diabetic foot surgeon who performs the operation must be an expert in distinguishing the different qualities of the bone [28,29]. The standardization of this practice, based on the evaluation of the density, anatomical structure, color, vascular thrombosis, and draining sinus will allow greater effectiveness in distinguishing between the healthy and infected bone [26] and will allow the reproducibility of the technique. The analysis of these parameters should be carried out by all operators who practice sequestrectomy of the infected bone. In this study, by standardizing the surgical practice and entrusting the treatment to an expert surgeon, we achieved eradication of the infected bone in 80% of cases. We recommend that all operators perform a double histological evaluation of the margin and the infected bone to validate their clinical practice.

The distinction between infected and healthy bone plays a key role in the treatment of osteomyelitis in the diabetic foot. Evidence shows that partial removal of the bone with an infected margin is fraught with an increased risk of further proximal amputation [27] and treatment failure despite a longer duration of antibiotic therapy [25]. Based on these data, the indication is to remove any areas of necrotic fragmented bone or areas of significant cortical destruction visible on X-ray and to continue until entirely healthy-looking bone is observed at the margin of resection [30].

Other authors have highlighted similar results in terms of healing regardless of the margin [31,32] but using long-term antibiotic therapies [32]. In our study, the presence of a positive margin found in five patients (20%) did not influence the healing outcome, but these cases presented a tendency to heal more slowly compared to those with the presence of a negative margin. An interesting observation is the finding of higher levels of acute inflammation and microbiological positivity of the infected bone in patients with a positive margin. These patients did not undergo further surgery given the absence of clinical signs of inflammation. The possible explanation for the favorable outcome can be given by the histological evidence: the positive margin showed significantly lower levels of acute inflammation compared to the corresponding infected bone.

Another fundamental point for the success of conservative treatment is represented by targeted antibiotic therapy. The choice to take the microbiological sample from the infected bone allowed us to obtain a high percentage of positive samples (80%) and to have a precise indication for the antibiotic therapy. After the outcome of the bone culture and given the high presence of resistant staphylococci and pseudomonas aeruginosa, we changed the antibiotic in 44% of patients to target the therapy. In other works, microbiological evaluation of the margin was performed compared with the clinically infected bone, obtaining lower percentages of positive cultures [33] and less possibility of directing antibiotic therapy. Furthermore, the epidemiological evaluation of these microbiological findings has prompted a change in the empirical approach, leading to increased use of tetracyclines and trimethoprim, which have demonstrated lower levels of resistance.

The average duration of antibiotic therapy was 21 days and was similar between subjects with a negative or positive margin. A prospective, randomized, and non-inferiority pilot study demonstrated that, after surgical debridement, a 3-week course of systemic antibiotic therapy for DFO resulted in an incidence of remission and adverse events that was similar and statistically non-inferior to a course of 6 weeks [34]. International guidelines recommend a duration of up to 3 weeks of antibiotic therapy after minor amputation for diabetes-related foot osteomyelitis and positive bone margin culture and a duration of 1–2 weeks if the infected bone is completely removed [11]. Data from other studies highlight longer durations of antibiotic therapy in clinical practice [32]. In our work, the duration of antibiotic therapy remains limited, and the absence of a significant difference between negative and positive margins may be motivated by the early start of antibiotic therapy while awaiting surgery, in particular if the osteomyelitis is associated with infection of soft tissues [5]. The effectiveness of this approach is confirmed by the high cure rate (80%) obtained and the absence of recurrence during follow-up. On the other hand, cases with a positive margin had a low level of residual inflammation, known as a key factor in achieving ulcer healing [35].

The strengths of our study are the combination of clinical, radiological, microbiological, histological, and surgical evaluations of osteomyelitis. This complex picture has been enriched by the possibility of carrying out and histologically demonstrating the effectiveness of a conservative surgical treatment associated with targeted antibiotic therapy, obtaining a high cure rate without relapses.

Our study has several limitations. The first limitation is that our study was retrospective. The second limitation is the small sample size. The third limitation is that we could not determine the role of anaerobic microorganisms in DFO. The final limitation of our study is that we included a white European population without racial/ethnic diversity, and, as a result, our results would benefit from testing in different populations.

## 4. Materials and Methods

### 4.1. Study Design and Participants

A single-center retrospective study was conducted at a tertiary institution from October to December 2022. The current study was a retrospective study using the administrative databases of the Monfalcone Hospital as the source of information. In this study, we analyzed patients with newly presenting osteomyelitis at our Foot Clinic. For this purpose, we observed all new lesions in the period between October and December 2022. The inclusion criteria were as follows: diabetic patients suffering from foot lesions with the presence of osteomyelitis. The exclusion criteria were as follows: absence of osteomyelitis and lesions involving a significant portion of the foot requiring amputation of the forefoot. We found 123 new lesions. A total of 82 lesions with Texas grade 1 or 2, without bone involvement, were therefore excluded from this study. Furthermore, 16 other lesions, Texas grade 3, were excluded as they affected a significant portion of the foot, requiring amputation of the forefoot. The remaining 25 lesions were included.

### 4.2. Clinical Diagnosis of DFO

The diagnosis of osteomyelitis was confirmed by a positive probe-to-bone test and a positive X-ray. The probe-to-bone test was performed with sterile metal forceps and was considered positive when the doctor could feel a sandy or hard surface. Two standard views of radiographs were obtained. If cortical collapse, periosteal ridge, sequestrum or involucrum, or gross bone destruction was noted, the patient was considered DFO-positive. Both tests had to be considered positive.

### 4.3. Treatment of DFO

After the clinical and vascular evaluation, the therapeutic procedure was planned, which involved the removal of the infected bone tissue. Ischemic patients were previously subjected to endovascular revascularization before the surgical procedure.

#### 4.3.1. Antibiotic Treatment

Upon presentation of the patient, in the presence of clinical signs of infection, empirical antibiotic treatment was started to resolve the inflammation of the soft tissues (36). Subsequently, based on the result of the bone culture obtained during surgery, the antibiotic therapy was simplified and targeted to the relevant bacteria.

#### 4.3.2. Surgical Intervention

After local anesthesia, the surgeon proceeded with the removal of the infected and non-viable soft tissues, isolated the bone segment affected by the osteomyelitis, and evaluated the clinical appearance of the bone. The infected bone has an altered consistency and appearance compared to healthy bone; this distinction is based on the evaluation of 5 parameters: density (hard vs. soft), anatomical structure (cortical integrity versus erosion, fracture), color (white versus black, gray), vascular thrombosis (open vessel vs. presence of clot), and draining sinus (pus in bone) [26]. Infected and necrotic tissues were first debrided. The resected infected bone was sent to the microbiology and pathology departments. After completely removing the infected bone, the surgeon changed their surgical gloves, and an additional bone specimen from the proximal margin was resected using a sterile Zaufal–Jansen or Luer forceps instrument. This was to ensure that the surgical instrument had not been previously used during the procedure and to avoid cross-contamination of the sample. The bone specimen resected from the proximal bone margin was collected for the pathology department. The surgeries were then completed with the remodeling of the residual bone and the closure, if possible, of the lesions.

#### 4.3.3. Outcome

The healing of the lesion was considered complete clinical resolution.

### 4.4. Sample Analysis

#### 4.4.1. Microbiological Analysis of Bone Samples

During the surgical procedure, after ulcer debridement, bone samples were collected from all patients using an aseptic technique [36]. Part of the bone sample believed to be infected was sent for microbiological analysis. Samples for culture examination were mechanically homogenized by the same microbiologist in 1 mL of sterile phosphate-buffered saline at pH 7.4 (PBS, Sigma Aldrich, St Louis, MO, USA) for 5 min. Subsequently, the homogenized samples were plated on culture medium and incubated at 35 °C for 48 h. Isolated microorganisms were identified via conventional Gram staining methods and classified as mono- or polymicrobial [28]. Susceptibility testing was performed in accordance with clinical and laboratory standards using the disk diffusion method [37].

#### 4.4.2. Histopathological Analysis of Bone Samples

The two histological samples, surgically determined infected bone and margin, from each patient were analyzed separately. The samples were decalcified, carved, embedded in paraffin, cut with the microtome, stained with hematoxylin–eosin, observed under a microscope, and classified as acute or chronic DFO [38]. Acute DFO was defined as multifocal infiltrate in the bone marrow of plasma cells, polymorphonuclear neutrophils and lymphocytes with clear polymorphonuclear predominance, and a variable proportion of concentrations of necrosis. Chronic DFO was defined as multifocal infiltrate in the bone marrow of plasma cells and lymphocytes with mononuclear predominance and some concentrations of reshaped bone with a variable fibrosis or osteoid formation [38]. We analyzed the presence of acute or chronic inflammation, assigning a grading with three levels: +, ++, +++. The operator was not aware of the type of bone taken.

### 4.5. Statistical Analysis

Statistical analysis was performed using the SPSS statistical package, version 12.0.2 (SPSS, Chicago, IL, USA). Continuous variables are described as the mean and SD, whereas categorical variables are reported as the percentage. The Student’s *t*-test was performed for quantitative variables distributed normally, and the Mann–Whitney U test was used for abnormally distributed quantitative parameters. *p*-values of <0.05 were considered significant with a 95% confidence interval.

### 4.6. Ethical Approval

The study was conducted in accordance with the 1964 Declaration of Helsinki and its later amendments. We used the general consent system signed by patients at first visit, in which they agree that clinical data can be used for clinical research, epidemiology, disease study, and training, with the aim of improving knowledge, treatment, and prevention.

## 5. Conclusions

In this study, we found that combining targeted antibiotic therapy with removal of the infected bone in the treatment of diabetic foot osteomyelitis is able to resolve the infection in a high percentage of patients. For this reason, a multidisciplinary team approach capable of ensuring all treatment steps is essential to reduce major and minor amputations and increase the chances of recovery [39]. Further studies with a larger number of cases are necessary to confirm these findings.

The resolution of osteomyelitis was also persistent without relapse. The removal of the infected bone, standardized based on an analysis of the density, anatomical structure, color, vascular thrombosis, and draining sinus, can be performed to differentiate the healthy bone from the infected bone. We demonstrated, from a histological point of view, that clinical differentiation corresponds to a change in bone quality, moving from acute to chronic inflammation. Last, but not least, the results obtained confirm the effectiveness of a multidisciplinary team led by diabetologists [40].

## Figures and Tables

**Table 1:** Patient characteristics.

Characteristics	Mean ± SD
Age	71 ± 10 years
Type 2 diabetes n (%)	25 (100%)
Male/female n	20/5
BMI (m^2^/kg)	27.4 ± 3.4
HbA1c (%)	7.9 ± 2.1
Creatinine (mg/dL)	1.09 ± 0.5
Peripheral neuropathy n (%)	25 (100)
Peripheral vascular disease n (%)	18 (72)
Ischemic disease n (%)	11 (44)
Arterial hypertension n (%)	25 (100)
Coronary artery disease n (%)	12 (48)
Cerebral vascular disease n (%)	5 (20)
Lesions Texas 3A n (%)	4 (16)
Lesions Texas 3B n (%)	8 (32)
Lesions Texas 3D n (%)	13 (52)
Evolution of the ulcers (days)	184 ± 198

**Table 2:** Resistance analysis of common bacteria to sensitive antibiotics.

	*Staphylococcus aureus* (5)	*Staphylococcus* *haemolyticus* (4)	*Pseudomonas* *aeruginosa* (4)
Oxacillin	3 (60%)	4 (100%)	
Quinolones	2 (40%)	1 (25%)	0
Clindamycin	2 (40%)	3 (75%)	
Tetracycline	0	1 (25%)	
Trimethoprim	0	1 (25%)	2 (50%)
Cephalosporin	3 (60%)	4 (100%)	1 (25%)
Erythromycin	3 (60%)	2 (50%)	

**Table 3:** Results of empiric antibiotics, bacteria, and targeted antibiotics correlated with healing and histology.

Sample	Soft Tissue Infection	Empiric Antibiotic	Microbiology	Bacterial	Targeted Antibiotic	Antibiotic Duration (Days)	Histology Infection	Healing
1	Yes	Amox/clav	Positive	*Citrobacter* *freundii*	Amox/clav	15	Positive	Yes
2	Yes	Amox/clav	Negative		Amox/clav	25	Positive	Yes
3	Yes	Amox/clav	Positive	*St. aureus*	Teicoplanin	14	Positive	Yes
4	Yes	Ciprofloxacin	Positive	*St. haemolyticus*	Moxifloxacin	25	Positive	Yes
5	Yes	Levofloxacin	Positive	*St. haemolyticus*	Levofloxacin	15	Positive	Yes
6	Yes	Amox/clav	Positive	*Clostridium* *sporogenes*	Amox/clav	20	Positive	Yes
7	Yes	Ciprofloxacin	Negative		Ciprofloxacin	15	Positive	Yes
8	Yes	Amox/clav	Positive	*Pseudomonas ae.*	Levofloxacin	15	Positive	No
9	Yes	Amox/clav	Positive	*Enterococcus* *faecalis*	Amox/clav	15	Positive	No
10	Yes	Amox/clav	Positive	*Citrobacter K* *St. aureus*	Levofloxacin	18	Negative	Yes
11	No	Levofloxacin	Negative		Levofloxacin	23	Positive	No
12	Yes	Pip/taz	Negative		Amox/clav	30	Positive	Yes
13	No		Negative			18	Positive	No
14	Yes	Pip/taz Teicoplanin	Positive	*Pseudomonas ae.*	Levofloxacin	45	Positive	Yes
15	Yes	Amox/clav	Positive	*Pseudomonas ae.*	Levofloxacin	17	Positive	Yes
16	Yes	Amox/clav	Positive	*St. aureus*	Levofloxacin	17	Positive	Yes
17	Yes	Pip/taz	Positive	*St. haemolyticus*	Levofloxacin Rifampicin	19	Positive	Yes
18	Yes	Amox/clav	Positive	*St. auricolaris*	Amox/clav	19	Positive	No
19	Yes	Amox/clav	Negative		Amox/clav	20	Negative	Yes
20	Yes	Amox/clav	Positive	*St. aureus*	Amox/clav	25	Positive	Yes
21	Yes	Levofloxacin	Positive	*St. haemolyticus*	Levofloxacin	20	Positive	Yes
22	Yes	Amox/clav	Positive	*Pseudomonas ae.*	Levofloxacin	20	Positive	Yes
23	Yes	Amox/clav	Positive	*Proteus mirabilis*	Amox/clav Levofloxacin	50	Positive	Yes
24	No	Trimet/sulf	Positive	*St. epidermidis*	Trimet/sulf	20	Positive	Yes
25	No	Levofloxacin	Positive	*St. aureus*	Levofloxacin	15	Positive	Yes

Abbr: *St.*: *Staphylococcus*, *ae.*: *Aeruginosa*, amox/clav: amoxicillin/clavulanate, pip/taz: piperacillin/tazobactam, trimet/sulf: trimethoprim/sulfamethoxazole.

**Table 4:** Histopathological aspects of infected bone and margin identified clinically.

		Infected Bone	Margin	*p*
Acute phlogosis	+	9	4	<0.001
	++	3	0	
	+++	7	1	
Chronic phlogosis	+	10	16	<0.001
	++	4	7	
	+++	0	0	
Microabscess		9	1	<0.001
Necrosis trabeculae		12	3	<0.001
Osteoclastic activity		2	5	=0.18

Presence of acute or chronic inflammation was graded in three levels: +, ++, +++.

**Table 5:** Comparison between the presence of infected and healthy margins.

	Negative Margin 20 Patients (80%)	Positive Margin 5 Patients (20%)	*p*
Age	73 ± 8.5	63 ± 9.7	<0.05
Male	75%	100%	n.s.
HbA1c	7.5 ± 2	10 ± 2	n.s.
Peripheral arterial disease	70%	80%	n.s.
BMI	27.5 ± 4.5	29.5 ± 2	n.s.
Creatinine (mg/dL)	1.2 ± 0.5	0.72 ± 0.1	<0.05
Infected bone phlogosis	1.15 ± 1	2.6 ± 0.8	<0.01
Positive microbiology	65%	100%	<0.01
Antibiotic duration	21 ± 8	23 ± 12	n.s.
Healing	75%	100%	n.s.
Time of healing (days)	63 ± 36	86 ± 51	n.s.

n.s.: not significant.

## Data Availability

The original contributions presented in this study are included in the article. Further inquiries can be directed to the corresponding author.
